# From Genomics to Precision Cardiology: A Comprehensive Review of Clinical Applications and Challenges in Cardiovascular Diseases

**DOI:** 10.1002/clc.70482

**Published:** 2026-10-03

**Authors:** Neda Mohsen‐Pour, Shiva Esmaeili, Aydin Feyzi, Ali Sharafi, Majid Maleki, Samira Kalayinia

**Affiliations:** ^1^ Zanjan Pharmaceutical Biotechnology Research Center Zanjan University of Medical Sciences Zanjan Iran; ^2^ Department of Molecular Medicine, Institute of Medical Biotechnology National Institute of Genetic Engineering and Biotechnology Tehran Iran; ^3^ Student Research Committee Shahid Beheshti University of Medical Sciences Tehran Iran; ^4^ Cardiogenetic Research Center Rajaie Cardiovascular Institute Tehran Iran

**Keywords:** cardiovascular genomics, genomic cardiology, multiomics integration, personalized cardiovascular therapy, polygenic risk score, precision medicine

## Abstract

**Background:**

Genomic cardiology is an emerging field integrating genetic, molecular, imaging, and digital health data to improve cardiovascular disease (CVD) prevention, diagnosis, and treatment. The genetic architecture of CVD encompasses rare, high‐impact variants as well as polygenic susceptibility, both of which influence disease risk, severity, and clinical outcomes.

**Hypothesis:**

Integration of genomic information, including rare variants and polygenic risk scores (PRS), with clinical, imaging, biomarker, and artificial intelligence (AI)‐based approaches may enhance personalized cardiovascular risk stratification and therapeutic decision‐making.

**Methods:**

This review summarizes current applications and emerging developments in genomic cardiology, including whole‐genome and whole‐exome sequencing, multigene panels, structural variant detection, PRS, multi‐omics integration, and AI‐assisted risk prediction. Current challenges and emerging therapeutic approaches, including RNA‐based therapies and gene editing, are also discussed.

**Results:**

Clinical genomic tools are increasingly being incorporated into the evaluation of inherited cardiac conditions. PRS can identify individuals with elevated lifelong CVD risk, although their current clinical utility is primarily as risk‐enhancing markers rather than standalone diagnostic tests. Integration of genomic, clinical, imaging, and biomarker data using AI may further improve risk prediction and personalized treatment selection. However, ancestry bias, uncertainty in variant interpretation, limited accessibility, workflow integration barriers, and ethical and privacy concerns remain important challenges.

**Conclusions:**

Genomic cardiology has substantial potential to advance precision cardiovascular medicine. Continued development of diverse genomic databases, standardized clinical implementation frameworks, and emerging genomic therapies will be important for translating genomic advances into equitable and clinically meaningful cardiovascular care.

## Graphical abstract

Genomic medicine in cardiovascular care is progressing from established diagnostic applications toward integrated risk prediction, multiomics, and emerging therapeutic strategies.

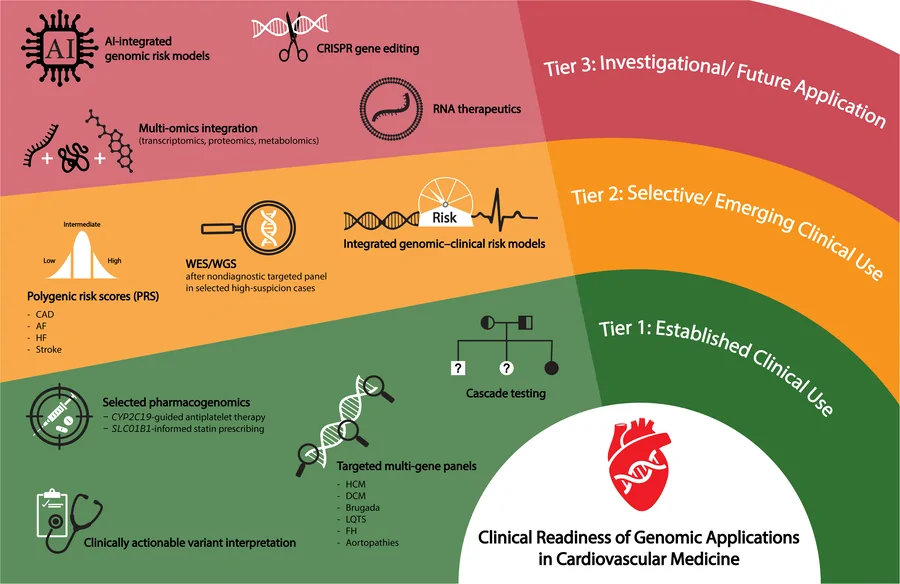

## 1 Introduction

Cardiovascular disease (CVD) remains the leading cause of global mortality and represents a major source of healthcare and socioeconomic burden worldwide. The disease encompasses a heterogeneous spectrum of disorders, including atherosclerotic disease, heart failure (HF), and cardiac arrhythmias, which arise from complex interactions between inherited genetic susceptibility, environmental exposures, and lifestyle‐related risk factors [1]. Population‐level and ancestry‐related genetic differences further contribute to variability in disease risk and clinical presentation [2, 3].

Despite advances in prevention and therapeutic management, CVD mortality has continued to increase over the past three decades, largely driven by population aging and persistent exposure to metabolic and behavioral risk factors. Ischemic heart disease (IHD) and stroke remain the dominant contributors to global cardiovascular mortality, particularly in Central and Eastern Europe and Central Asia [4]. Moreover, cardiovascular, and metabolic disorders share overlapping biological pathways and social determinants, amplifying the worldwide disease burden [5, 6, 7, 8, 9].

Clinical outcomes in advanced CVDs remain suboptimal for certain patient groups, especially individuals with hereditary cardiomyopathies or treatment‐resistant HF, where therapeutic options are often limited to cardiac transplantation despite challenges related to donor availability and post‐transplant complications [10, 11, 12]. These clinical limitations highlight the need for precision therapeutic strategies that target disease mechanisms rather than solely phenotypic manifestations.

Genomic medicine has emerged as a promising framework for precision cardiovascular care by enabling the identification of pathogenic variants that can inform disease diagnosis, risk stratification, prognostic assessment, and family‐based screening strategies. However, a substantial proportion of rare variants, particularly missense variants, remain classified as variants of uncertain significance (VUS) due to insufficient functional or population‐level evidence [13]. The rapid expansion of multiomics technologies and high‐throughput sequencing platforms, including whole‐genome sequencing (WGS), whole‐exome sequencing (WES), and targeted sequencing approaches, has accelerated cardiovascular genetic discovery but has also introduced new challenges in biological interpretation and clinical translation [14, 15]. Large multi‐ancestry biobank resources are increasingly recognized as essential infrastructures for precision medicine research, enabling more accurate characterization of genotype–phenotype relationships across diverse populations. Together, these developments collectively support a shift in cardiovascular research from population‐level risk assessment to mechanism‐oriented and patient‐specific therapeutic strategies.

This review aims to synthesize current evidence regarding the role of genomics in CVD, with a particular emphasis on clinical applications of precision cardiology. Specifically, the manuscript focuses on how genetic and genomic insights contribute to understanding molecular disease mechanisms, improving diagnostic strategies, and facilitating personalized cardiovascular treatment approaches. Despite rapid advances in cardiovascular genomics, several critical knowledge gaps remain. First, the clinical utility of many genomic discoveries is still limited by incomplete functional validation and uncertainty in variant interpretation, particularly for rare missense variants and VUS. Second, most genomic association studies have been conducted in populations of European ancestry, limiting the generalizability of risk prediction models and polygenic risk scores (PRS) across diverse populations. Third, translation of genomic findings into routine cardiovascular practice remains incomplete, as robust evidence linking genomic testing to improved hard clinical outcomes is still limited. Addressing these gaps is essential for advancing mechanism‐based precision cardiology and achieving equitable clinical implementation of genomic medicine. Unlike many earlier reviews that primarily focused on isolated aspects of cardiovascular genomics, this manuscript integrates molecular genetic architecture, clinical translation, multiomics convergence, and emerging therapeutic paradigms within a single comprehensive framework. Given the rapidly expanding and highly heterogeneous landscape of cardiovascular genomics, the present review is structured around a three‑tier framework based on clinical readiness. Tier 1 comprises genomic applications supported by professional guidelines and already integrated into routine cardiovascular practice. Tier 2 encompasses approaches that have shown promise and may offer clinical utility in selected patient subsets, but that require further prospective validation before widespread adoption. Tier 3 covers investigational technologies that remain predominantly experimental and are not yet ready to influence routine clinical management. This framework is designed to assist practicing cardiologists in navigating the spectrum from established genomic tools to emerging precision medicine strategies, while preserving a clear distinction between clinical actionability and scientific promise.

## 2 Methodology

This narrative review summarizes current evidence on cardiovascular genomics and precision cardiology. Literature searches were conducted in PubMed, Scopus, and Google Scholar for publications between January 2015 and January 2026. Searches were performed using combinations of the following keywords: CVD, genomics, rare and common genetic variation, genome‐wide association studies (GWAS), PRSs, WES, WGS, and multiomics approaches. Representative search strings included terms such as cardiovascular genomics, inherited cardiac conditions, genetic testing guidelines, and PRSs in CVDs. To identify major guidelines and consensus statements, we conducted targeted searches of professional society websites (European Society of Cardiology, American Heart Association, Heart Failure Society of America, and American College of Medical Genetics and Genomics) and manually screened the reference lists of retrieved articles and relevant reviews.

Priority was given to large population studies, multi‐ancestry genomic research, clinically relevant translational investigations, and, where available, prospective studies, randomized trials, implementation studies, guidelines, and large biobank analyses when making clinical claims. Studies published after 2020 were emphasized to reflect recent developments in genomic technologies. Article selection was based on clinical relevance, methodological quality, sample size, population diversity, and contribution to precision cardiology research. Two authors independently screened titles and abstracts, and full‐text assessment was performed when necessary. Disagreements in article selection were resolved through consensus discussion among the authors. Reference lists of key articles were also manually checked.

As this is a narrative review, no formal meta‐analysis or systematic‐review methodology was performed. Evidence was synthesized qualitatively according to study design, biological relevance, and clinical applicability. The objective of the methodology was to provide a clear and clinically meaningful overview of genomic advances in cardiovascular medicine rather than exhaustive systematic aggregation.

## 3 Genetic Architecture of Cardiovascular Diseases

### 3.1 Monogenic Versus Polygenic Cardiovascular Diseases

Cardiovascular diseases can be broadly classified into monogenic and polygenic disorders. Monogenic CVDs result from pathogenic variants in a single gene that affect protein structure or function. Examples include hypertrophic cardiomyopathy (HCM), dilated cardiomyopathy (DCM), arrhythmogenic cardiomyopathy (ACM), channelopathies such as long‐QT syndrome (LQTS) and catecholaminergic polymorphic ventricular tachycardia, aortopathies, and familial hypercholesterolemia (FH) [16, 17]. Although traditionally considered Mendelian disorders, monogenic CVDs often show incomplete penetrance and phenotypic heterogeneity due to environmental factors, lifestyle influences, and co‐inherited common variants [18, 19].

Polygenic CVDs arise from the cumulative effect of multiple common variants, typically located in non‐coding genomic regions. Examples include hypertension, atrial fibrillation (AF), and coronary artery disease (CAD). Genome‐wide association studies have identified numerous cardiovascular susceptibility loci over the past two decades [16, 20, 21].

Polygenic risk scores (PRS), derived from GWAS data, aggregate multiple single‐nucleotide polymorphisms (SNPs) to quantify inherited susceptibility. PRS can improve risk prediction beyond conventional clinical factors and support early prevention and personalized intervention strategies [22, 23, 24]. In CAD and cardiomyopathies, PRS also modulate disease expression in carriers of monogenic pathogenic variants [25].

Cardiovascular disease is a highly heritable polygenic disorder, with more than 160 susceptibility loci reported in large‐scale GWAS [3]. However, routine clinical implementation of PRS remains limited due to interpretational uncertainty and reduced predictive performance in non‐European populations [26, 27]. However, while PRS can stratify relative cardiovascular risk, their absolute predictive value and clinical actionability remain debated. Improvements in statistical prediction or risk reclassification do not necessarily equate to clinically meaningful benefit. Evidence that PRS‐guided interventions improve hard cardiovascular outcomes in large, diverse populations remains limited, and most data are derived from observational studies rather than randomized controlled trials.

### 3.2 Common Versus Rare Variants

Cardiovascular diseases are influenced by both common and rare genetic variants, which contribute through complementary biological mechanisms. Rare variants usually have large individual effects but are present in a small proportion of patients, whereas common variants have modest individual effects but collectively shape polygenic susceptibility [23].

Heart failure genetics has been extensively studied using large‐scale sequencing approaches. Lee and colleagues analyzed common and rare variants in more than 2.3 million individuals and identified 176 common‐variant risk loci. These loci were organized into modules associated with obesity, blood pressure regulation, atherosclerosis, immune pathways, and arrhythmogenesis. Exome sequencing of 376 334 individuals demonstrated significant associations between HF and rare loss‐of‐function variants in *TTN*, *MYBPC3*, *FLNC*, and *BAG3*. Rare coding variants mainly contributed to risk through Mendelian cardiomyopathy genes, whereas common‐variant heritability was distributed across the genome. Importantly, a polygenic background can modify disease penetrance in carriers of rare pathogenic variants. For example, common‐variant risk modifies the clinical expression of truncating *TTN* variants, suggesting that monogenic testing alone may not fully capture HF genetic risk [28]. While these findings highlight the potential of integrated genomic approaches, the clinical utility of combining rare variant analysis with polygenic risk assessment for guiding individual patient management remains to be established. Prospective validation in clinical settings is needed before these approaches can be routinely implemented.

Compound heterozygous variants in *TTN* were reported in a consanguineous family with Salih myopathy and DCM, confirming the contribution of rare recessive variation to cardiac muscle disease [29]. Pathogenic variants in *SCN5A* are associated with multiple inherited cardiac disorders, including Brugada syndrome and LQTS. These variants may also produce overlap phenotypes involving cardiac conduction abnormalities and ventricular arrhythmias [30].

In patients with CAD, rare DCM‐related variants, including *TTN*, combined with high polygenic risk were associated with reduced left ventricular function and subclinical structural abnormalities [31]. Exome‐wide association analysis identified rare damaging variants in *HTRA1*, *SGTB*, and *RBM12* that increased atherosclerosis risk independent of conventional cardiovascular risk factors [32].

GWAS of bradyarrhythmia involving 1.3 million individuals identified multiple susceptibility loci related to ion channel biology, cardiac development, and cellular homeostasis. Both common and rare variants in genes such as *SCN5A*, *SCN10A*, *LMNA*, *MYBPC3*, and *TTN* contribute to sinus node dysfunction, conduction disease, and pacemaker implantation risk [33].

Variants in G‐protein‐coupled receptor genes involved in gut–host metabolic signaling, including *FFAR3*, *FFAR2*, and *HCAR2*, were associated with hypertension and major adverse cardiac events, suggesting a role for microbiome‐related signaling pathways in cardiovascular risk [34]. Overall, CVD genetic architecture exists along a continuum ranging from monogenic high‐impact variants to highly polygenic susceptibility. Rare pathogenic variants identify individuals at very high risk, whereas polygenic background modulates penetrance, expressivity, and clinical severity. Integrating rare variant analysis with polygenic risk assessment may improve precision cardiovascular risk stratification and guide personalized prevention and therapy. Table 1 summarizes the key characteristics of cardiovascular genetic architecture across major disease categories. Figure 1 illustrates the conceptual framework of this continuum.

**Table 1:** Summary of genetic architecture and clinical relevance of major cardiovascular diseases in precision cardiology.

Disease category	Predominant variant classes	Representative genes/loci (examples)	Clinical utility of genetic information	Clinical management implications	Key references
CAD	Common noncoding SNPs; some rare coding variants	9p21, *PCSK9*, *LDLR*, *APOB*, *LPA*	Risk prediction, prevention strategies, guidance for lipid‐lowering therapy	High‑risk variants guide statin intensity; FH variants prompt cascade screening and *PCSK9* inhibitor consideration	[35, 36, 37]
HF	Common SNPs; rare LoF variants	*TTN, MYBPC3, BAG3, FLNC*	Etiologic classification, prognosis, family screening	*TTN*tv carriers warrant family screening; *LMNA* variants prompt early ICD evaluation	[17, 28]
HCM	Rare missense/LoF variants; common SNPs	*MYH7, MYBPC3, TNNT2*	Diagnosis, penetrance stratification, prognostic refinement	Pathogenic variants guide ICD decisions and sports restriction; polygenic score may refine penetrance estimates	[17, 38]
DCM	Rare truncating variants; common SNPs	*TTN, LMNA, FLNC, BAG3*	Diagnosis, risk stratification, cascade testing	*LMNA*, *FLNC*, *RBM20* variants prompt early ICD consideration; cascade testing for relatives	[28, 31]
Cardiac arrhythmias (AF, Brugada, LQTS)	Common SNPs; rare coding variants	*SCN5A, SCN10A, KCNQ1*	Risk prediction, diagnosis, therapy selection	*SCN5A* variants guide sodium channel blocker use; *KCNQ1*/*KCNH2* variants guide beta‑blocker and lifestyle advice	[30, 39]
Aortopathies/connective tissue disorders	Rare pathogenic variants	*FBN1, TGFBR1, TGFBR2, COL3A1, ACTA2*	Diagnosis, surveillance, surgical timing, family screening	Gene‑specific surveillance intervals; *COL3A1* prompts vascular imaging; *FBN1* guides prophylactic aortic surgery	[40, 41]
CHD	Rare coding and non‐coding variants	*NOTCH1, GATA4, TBX5, NKX2‐5*	Etiologic diagnosis, recurrence risk counseling	Genetic diagnosis guides syndromic evaluation; informs recurrence risk for family planning	[42, 43]
Stroke/hypertension	Common SNPs with pleiotropy	*SH2B3, PTPN11, ALDH2*	Risk stratification, prevention	PRS may refine risk; *ALDH2* variants guide alcohol avoidance in East Asian populations	[44, 45]

**Figure 1:**
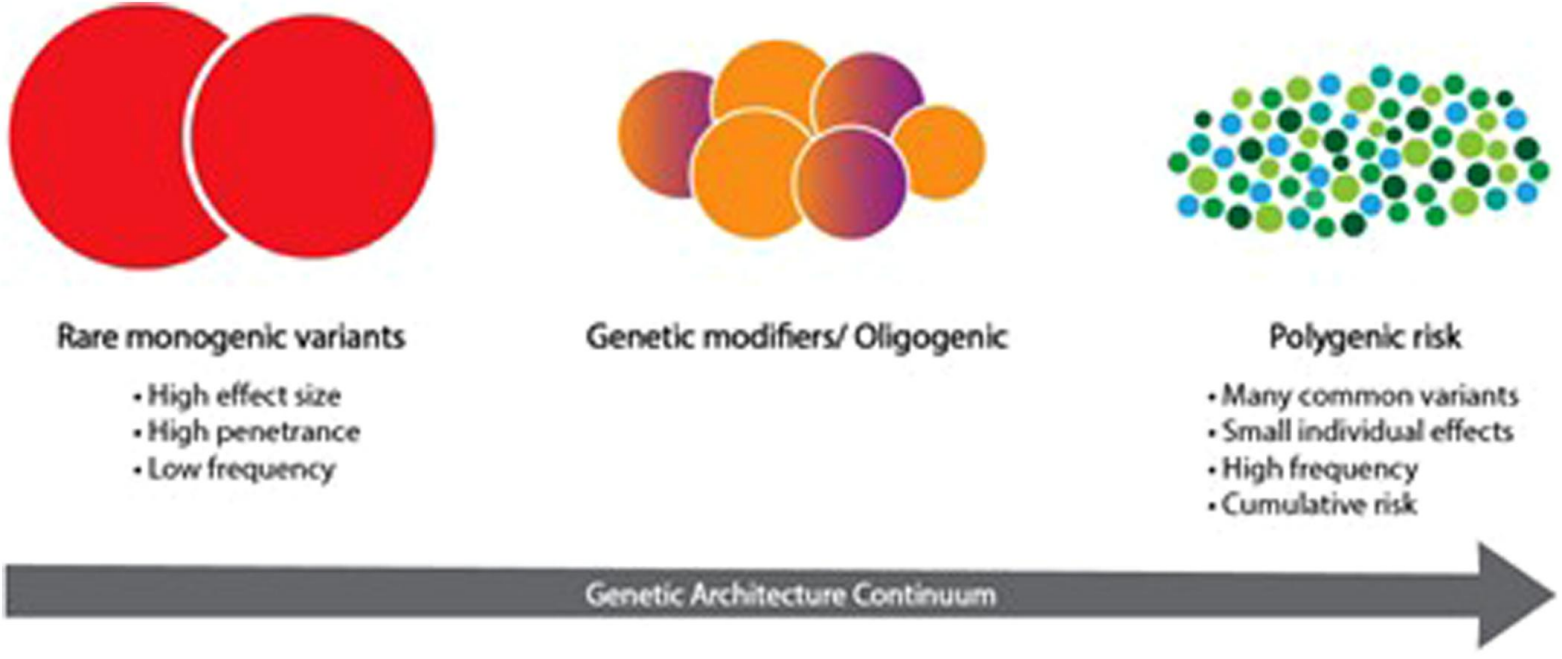
Illustration of the continuum of cardiovascular genetic architecture from monogenic high‐impact variants to highly polygenic susceptibility models. Rare pathogenic variants typically exert strong biological effects and are associated with high penetrance but low population frequency. In contrast, polygenic architecture results from the cumulative influence of numerous common variants with individually modest effects that collectively modulate disease risk, penetrance, and clinical expression. The framework highlights the role of genetic modifiers and supports integrating rare variant analysis with polygenic risk assessment for precision cardiovascular risk stratification.

### 3.3 Insights From GWAS and Post‐GWAS

The genetic basis of CVDs has been extensively investigated, particularly following the completion of the Human Genome Project and advances in genomic technologies. A major milestone in this field was achieved in 2007 with the first GWAS, which identified more than 300 loci associated with CAD and 89 partially overlapping loci associated with stroke [35, 46, 47, 48]. These discoveries demonstrated that both CAD and stroke have a polygenic architecture, characterized by numerous alleles distributed across the genome, each contributing a modest effect to disease susceptibility [49]. Since then, thousands of genetic variants have been reported to be associated with a wide spectrum of CVD phenotypes [35, 47, 50, 51, 52, 53, 54]. Current research in this area follows two complementary strategies to advance understanding of CVD genetics. The first strategy focuses on increasing the statistical power of genome‐wide analyses by expanding cohort sizes, incorporating underrepresented populations and ancestries, and interrogating rare variants through WES and WGS [55, 56, 57]. The second strategy aims to translate GWAS findings into personalized medicine applications, including the identification of therapeutic targets, opportunities for drug repurposing, and the development of PRSs to identify individuals at elevated risk of disease [49, 58, 59]. Although individual variants identified by GWAS generally exert small effects, their cumulative contribution explains a substantial proportion of disease heritability [60]. GWAS identify genetic variants associated with CVDs by scanning the genomes of large populations to detect markers linked to disease‐related traits. Through this approach, numerous loci have been implicated in the risk and progression of cardiovascular conditions. Notably, significant associations have been reported between SNPs and disorders such as CAD, myocardial infarction (MI), and AF [35, 39, 61, 62]. Beyond independent effects of rare and common variants, emerging evidence indicates that polygenic background substantially modifies the penetrance and clinical expression of monogenic CVDs (Table 2).

**Table 2:** Interaction between rare pathogenic variants and polygenic background across cardiovascular phenotypes.

Cardiovascular phenotype	Rare pathogenic variant(s)	Polygenic background	Observed clinical effect	Clinical management implications	Key references
HF	*TTN* truncating variants	HF PRS	Increased HF risk and severity; polygenic background modifies penetrance	Cascade testing for at‑risk relatives; regular LVEF monitoring; early referral for HF management	[28]
HCM	*MYH7, MYBPC3* variants	HCM PRS	Up to tenfold difference in disease penetrance and outcomes	Consider early surveillance; cascade testing for at‑risk relatives	[38]
DCM with CAD	*TTN* and other DCM variants	DCM PRS	Lower LVEF and subclinical LV dysfunction	PRS may identify subclinical DCM in CAD patients; early imaging follow‑up; does not yet alter ICD decisions	[31]
Inherited arrhythmias	*SCN5A* pathogenic variants	Arrhythmia‐related common variants	Broad phenotypic spectrum and overlap syndromes	Variant‑specific therapy (e.g., sodium channel blockers); cascade testing	[30]
Bradyarrhythmia	*LMNA, SCN5A, TTN*	Common conduction‐related loci	Increased risk across sinus node dysfunction and pacemaker implantation	*LMNA* carriers: early EPS and ICD evaluation	[33]
Atherosclerotic disease	*HTRA1, SGTB* rare variants	Polygenic CAD background	Elevated CAD risk independent of traditional factors	Enhanced risk stratification; consider earlier lipid‑lowering therapy	[32]
Hypertension/MACE	GPCR rare variants (*FFAR3, FFAR2*)	Cardiometabolic PRS	Increased hypertension and HF risk	Blood pressure optimization; monitor for HF progression	[34]

These observations collectively emphasize the spectrum of genetic influences in CVDs, spanning rare high‐impact variants and distributed polygenic susceptibility, supporting integrated genomic approaches for improved cardiovascular risk stratification and clinical translation. Cardiovascular genetics spans a continuum from monogenic to polygenic risk. While GWAS have substantially expanded our knowledge, clinical translation remains limited by ancestry bias and insufficient prospective validation. The principal challenge is integrating these insights into actionable clinical workflows.

### 3.4 Coronary Artery Disease and Inherited Dyslipidemias

Coronary artery disease results from atherosclerosis, a complex process involving multiple cell types that leads to lipid‐rich plaque formation and impaired coronary blood flow. GWAS and WGS have identified numerous variants associated with CAD and its risk factors, including blood pressure, lipid traits, type 2 diabetes, and smoking. Interpretation of noncoding regulatory variants remains challenging because of linkage disequilibrium patterns [63].

Population genomic studies have revealed shared and heterogeneous genetic risk across CAD‐related phenotypes. For example, Koyama and colleagues identified an association between CAD and a missense variant in *RNF213*, a gene also linked to Moyamoya disease, using WGS data from Japanese cohorts [64]. However, the generalizability of this association across ancestries remains uncertain [3]. Large multi‐ancestry GWAS analyses have identified novel susceptibility loci and highlighted ancestry‐dependent performance of PRS. For example, Tcheandjieu et al. identified 95 novel loci associated with CAD, with 9p21 haplotypes showing risk stratification across populations except African ancestry groups, demonstrating reduced PRS transferability [36]. Despite these advances, the clinical utility of PRS for CAD in routine practice remains uncertain. Small improvements in discrimination or reclassification do not necessarily establish that PRS‐guided treatment improves cardiovascular outcomes. Prospective trials comparing PRS‐guided management with conventional risk assessment tools are lacking.

The meta‐genomic risk score (metaGRS), integrating genome‐wide and targeted genetic variants, further improved CAD risk stratification. In a cohort of more than 480,000 UK Biobank participants, individuals in the top risk percentile showed substantially higher CAD risk compared with conventional clinical risk factors [37]. Functional genomic analyses have identified genes involved in lipid metabolism, vascular remodeling, and pleiotropic signaling pathways. Bayesian network modeling using GWAS summary data revealed 142 CAD‐associated genes, including canonical loci such as *OLIG1* and pleiotropic genes such as *TCF7* [65]. Approximately 42%–47% of CAD‐associated variants appear to act independently of traditional cardiovascular risk factors [35, 36]. Understanding this architecture can improve prediction, prevention, and treatment strategies [63].

Lipidomics–genomics integration studies further support the mechanistic link between lipid metabolism and atherosclerosis. Residualized apolipoprotein B (apoB) levels were associated with MI risk, suggesting genetic regulation of lipid pathways beyond standard LDL‐C measurement [66]. Spontaneous coronary artery dissection (SCAD) represents a distinct genetic and mechanistic subtype of coronary disease. GWAS meta‐analysis identified 16 susceptibility loci, including genes related to vascular smooth muscle and extracellular matrix biology. The tissue factor gene *F3* showed SCAD‐specific association, and some loci demonstrated opposite effects compared with atherosclerotic CAD. Hypertension was also identified as a causal risk factor [67].

Polygenic risk models for dyslipidemia further demonstrate phenotype‐specific effects. LDL‐specific PRS was associated with plaque rupture, severe coronary stenosis, thrombotic CAD, and mortality, whereas triglyceride‐specific PRS showed a stronger association with thrombotic coronary disease [68]. In heterozygous familial hypercholesterolemia (HeFH) patients, CAD‐specific PRS predicted approximately twofold higher atherosclerotic CVD risk independent of clinical variables [69].

Across ancestry groups, genetically predicted LDL‐C levels were consistently associated with CAD risk, although effect sizes varied, reflecting population heterogeneity [70]. Mendelian randomization analyses further supported causal associations between genetically predicted apoB, LDL‐C, and CAD outcomes, while triglyceride effects showed potential pleiotropy [71]. Genetic predisposition to elevated lipoprotein(a) [Lp(a)] was also associated with obstructive coronary plaque burden independent of LDL‐C [72]. Overall, CAD represents a heterogeneous disorder driven by interactions between lipid metabolism, vascular biology, and polygenic susceptibility. Integrating rare high‐impact variants, polygenic risk models, and functional genomic data supports personalized prevention and precision cardiovascular management.

### 3.5 Inherited Arrhythmias and Cardiomyopathies

Inherited arrhythmias and cardiomyopathies exhibit a combined monogenic and polygenic genetic architecture. Rare pathogenic variants in genes such as *SCN5A*, *MYH7*, and *MYBPC3* represent major monogenic contributors to diseases including Brugada syndrome, HCM, DCM, LQTS, and related conduction disorders [17].

Common genetic variation also plays an important modifier role. GWAS‐derived polygenic scores significantly influence disease susceptibility, penetrance, and phenotypic expression. For example, Zheng et al. demonstrated that higher polygenic scores were associated with increased HCM risk and could produce up to tenfold differences in disease penetrance between genetic risk strata [38]. While these findings provide valuable mechanistic insights, their immediate clinical actionability is limited. The extent to which polygenic scores can guide clinical management, such as decisions regarding implantable cardioverter‐defibrillator (ICD) or surveillance intervals, remains unclear and requires prospective validation.

### 3.6 Aortopathies and Connective Tissue Disorders

Hereditary thoracic aortic diseases (HTADs) are clinically, and genetically heterogeneous disorders characterized by progressive aortic wall degeneration due to connective tissue abnormalities. Syndromic connective tissue disorders, including Marfan syndrome, Loeys–Dietz syndrome (LDS), vascular Ehlers–Danlos syndrome (vEDS), and related conditions, are major contributors to inherited aortopathy. These disorders typically present with early‐onset aortic dilation, aneurysm formation, and dissection risk [40].

Advances in next‐generation sequencing (NGS), including targeted gene panels and WES, have identified pathogenic variants in *FBN1*, *TGFBR2*, and *SLC2A10*, correlating with Marfan syndrome, Loeys–Dietz syndrome, and arterial tortuosity syndrome, respectively [73]. Inherited aortopathies disrupt extracellular matrix integrity, smooth muscle contractility, and TGF‐β signaling pathways. Pathogenic variants in *FBN1*, *TGFBR1*, *TGFBR2*, *COL3A1*, *ACTA2*, and *MYH11* contribute to both syndromic and non‐syndromic thoracic aortic disease [41]. Clinical presentation and disease progression vary by genotype. For example, Yagyu and colleagues showed that variants in the TGF‐β pathway were associated with higher subsequent cardiovascular event rates compared with *FBN1* variants. Patients carrying *COL3A1* variants experienced predominantly extra‐aortic arterial complications, with sex‐dependent differences in clinical expression [74]. Similarly, arterial events occurred earlier and more frequently in *COL3A1*‐related vEDS compared with other HTAD subtypes. Extra‐aortic arterial aneurysms are also common in Loeys–Dietz syndrome, highlighting the need for comprehensive vascular imaging and longitudinal surveillance [75, 76].

### 3.7 Congenital Heart Disease: From Monogenic to Oligogenic Inheritance

Genetic and genomic testing plays an important role in patients with congenital heart disease (CHD) and cardiomyopathies. Identification of chromosomal abnormalities, copy number variants (CNV), and monogenic mutations supports prognosis, treatment planning, and family risk assessment. Despite relatively high diagnostic yield, genetic testing remains underutilized, highlighting the need for standardized clinical protocols and improved access to genomic resources [77].

Congenital heart disease represents a heterogeneous group of structural cardiac defects that present at birth, with syndromic and familial forms contributing to complex genetic architecture. Monogenic variants account for less than one‐third of cases. In the 100,000 Genomes Project, approximately 5.11% of participants initially received pathogenic or likely pathogenic findings. Application of a de novo Gene Discovery Pipeline increased diagnostic yield by about one‐third and identified additional pathogenic variants, including 79 candidate CHD genes such as *AKAP13*, *BCAR1*, *TFAP2C*, and *SETDB1* [42]. This supports the utility of comprehensive genome sequencing combined with agnostic variant discovery. Despite these promising findings, the clinical utility of comprehensive genome sequencing for routine CHD management remains uncertain. The diagnostic yield (approximately 5%‐10%) remains modest, and the cost‐effectiveness of broad genomic screening in this population has not been established.

Rare recessive variation also contributes to CHD pathogenesis. Baird et al. analyzed 49 patients from consanguineous Pakistani families and identified 758 rare homozygous protein‐altering variants across 693 genes. Prioritized candidates included 23 high‐confidence genes enriched for recessive CHD‐associated loci in mouse models. Cilia‐related genes were overrepresented, and functional validation of *ADCY6* confirmed its role in cardiac development [78].

Noncoding variants also play an important role in CHD susceptibility. Meta‐analyses involving more than 55 000 individuals demonstrated moderate‐to‐strong effects on phenotypes including atrial septal defect, conotruncal heart disease, left ventricular outflow tract obstruction, and complex cardiac malformations. Many variants influence chromatin organization and three‐dimensional genomic regulation of cardiac developmental genes such as *DLG1*, *BDH1*, and *SPAG9*, supporting oligogenic and phenotype‐specific inheritance patterns [43].

Clinical genetic evaluation in critically ill infants with CHD shows diagnostic yields comparable to critically ill infants without CHD. While karyotyping and chromosomal microarray analysis remain standard diagnostic approaches, WES provides additional diagnostic value, particularly for variants not detected by targeted panels. Molecular diagnostic rates are higher in atrioventricular and ventricular septal defects, whereas extracardiac anomalies such as esophageal atresia are associated with lower yield, emphasizing the importance of early systematic genetic testing for personalized clinical management [79].

Collectively, hereditary aortopathies and connective tissue disorders exhibit marked genetic and phenotypic heterogeneity. Pathogenic variants in genes involved in extracellular matrix structure, transforming growth factor‐β (TGF‐β) signaling, collagen synthesis, and smooth muscle contractility, including *FBN1*, *TGFBR1*, *TGFBR2*, *COL3A1*, *ACTA2*, and *MYH11*, contribute to variable risks of aortic aneurysm, dissection, and systemic vascular involvement. Gene‐specific effects influence age of onset, vascular distribution, and disease severity, supporting individualized surveillance, imaging strategies, and prophylactic intervention.

Similarly, CHD shows monogenic, oligogenic, and noncoding regulatory variant contributions with high phenotype specificity. Comprehensive genome sequencing and functional variant interpretation are therefore essential for early diagnosis, risk stratification, and family counseling, underscoring genomics' central role in cardiovascular precision medicine.

### 3.8 Polygenic Hypertension, Stroke and Shared Cardiometabolic Risk Architecture

Large‐scale genomic analyses demonstrate shared polygenic architecture between stroke and cardiometabolic risk factors, particularly through lipid metabolism pathways. Bai and colleagues identified 147 pleiotropic loci jointly associated with lipid traits and stroke, along with 10 shared genes, including *CUX2*, *SH2B3*, and *ICA1L*. Colocalization analyses suggested that many loci are driven by shared causal variants, with enrichment of *SH2B3* and *PTPN11* in adipose, musculoskeletal, and brain tissues. Mendelian randomization analyses supported the protective role of high‐density lipoprotein cholesterol (HDL‐C) and demonstrated that elevated triglyceride levels increase ischemic stroke risk. Transcriptome‐wide association analysis further identified *ALDH2* as a pleiotropic gene linking lipid metabolism to stroke susceptibility, supporting a shared cardiometabolic–cerebrovascular genetic framework [44].

PRSs improve prediction of cardiometabolic disease susceptibility. O'Sullivan and colleagues evaluated PRS performance across seven conditions, including CAD, stroke, hypertension, HF, cardiomyopathies, obesity, AF, and type 2 diabetes. PRS integration with clinical risk models improved prediction for CAD, AF, and type 2 diabetes, although predictive utility remained limited for ischemic stroke and hypertension. Expanding GWAS data sets and increasing population diversity may enhance PRS generalizability [45]. Similarly, Huang and colleagues assessed ischemic stroke PRS combined with disease specific PRS for AF, venous thromboembolism, CAD, hypertension, and type 2 diabetes in 480,000 UK Biobank participants over 12.5 years. Both composite and disease‐specific PRS were independently associated with incident stroke, and incorporation of PRS into clinical models modestly improved risk stratification, with some individuals reclassified from low‐ to high‐risk categories [80].

PRS performance varies across ancestral populations. Kember et al. evaluated multi‐ancestry PRS models for CAD, hypertension, type 2 diabetes, and body mass index in African‐ and European‐ancestry cohorts. PRS showed stronger predictive performance in European populations, reflecting underlying GWAS sampling bias. Incorporation of multi‐ancestry linkage disequilibrium panels improved prediction accuracy in African ancestry individuals. Phenome‐wide analyses further demonstrated shared genetic correlations across cardiometabolic comorbidities, emphasizing the importance of globally representative genomic data sets for equitable risk prediction [81].

Polygenic susceptibility also influences therapeutic response. Huo et al. examined treated hypertensive adults stratified by polygenic risk for systolic blood pressure. Individuals in the high‐risk genetic category exhibited higher systolic blood pressure, poorer hypertension control, and increased stroke hazard despite pharmacological therapy. These findings support incorporating polygenic risk information into hypertension management strategies to address residual stroke risk [82].

Population‐specific genetic correlation patterns have also been observed. Yamamoto et al. analyzed GWAS data sets from 232 987 East Asian and 751 051 European individuals to investigate genetic correlations between respiratory and cardiometabolic disorders. Genetic correlation directionality differed by population, being negative in East Asian cohorts and positive in European cohorts. Pathway analyses implicated peroxisome proliferator‐activated receptor signaling, suggesting that immune regulation and lipid metabolic pathways contribute to multimorbidity patterns across populations [83]

Collectively, these studies indicate that polygenic risk contributes to both hypertension and stroke susceptibility through shared cardiometabolic pathways. Integration of PRS with clinical risk factors enhances predictive performance, although ancestry‐related transferability bias remains a major limitation. Future research should prioritize multi‐ethnic GWAS cohorts and multi‐trait polygenic modeling to support precision cardiovascular risk assessment and equitable implementation of genomic medicine.

Despite rapid progress in polygenic modeling, major challenges remain in clinical implementation, including ancestry‐specific prediction bias, limited prospective validation, and incomplete integration of genomic risk into routine cardiovascular care pathways.

## 4 Genomic Tools for Precision Cardiology

Genomic tools enable personalized cardiovascular risk assessment, diagnosis, and therapy by integrating rare and common variant information across CVDs [84]. Core technologies include sequencing approaches for variant detection, polygenic risk modeling, clinical–genetic integration, pharmacogenomics, and emerging molecular therapies [85].

### 4.1 Whole Genome and Exome Sequencing

WES and WGS are increasingly used in the diagnostic evaluation of inherited cardiac conditions (ICC), including cardiomyopathies, primary arrhythmia syndromes, and familial aortopathies [86]. WES efficiently captures coding variants, where most established pathogenic mutations are located, whereas WGS enables broader detection of non‐coding variants, splice‐site alterations, mitochondrial variants, and structural rearrangements [87]. WGS also improves identification of deep intronic and cryptic splice variants that may be missed by targeted panel testing [86].

Diagnostic yield of WES/WGS varies across phenotypes, being highest in classic sarcomeric hypertrophic cardiomyopathy (approximately 30%–60%) and lower in dilated cardiomyopathy (10%–40%), ACM (10%–50%), and LQTS (50%–75%) [88, 89]. Sequencing results support disease confirmation, subtype classification, and cascade family screening. A major limitation remains the high prevalence of VUS, particularly in understudied ancestral populations [86]. Clinical interpretation of sequencing data requires multidisciplinary ICC teams involving cardiologists, clinical geneticists, genetic counselors, and bioinformatics specialists [90]. These teams coordinate test selection, pre‐ and post‐test counseling, and reporting of primary and secondary findings. As sequencing costs continue to decrease, WGS is increasingly considered a comprehensive genomic testing approach in tertiary precision cardiology centers [84].

### 4.2 Targeted Multigene Panels

Targeted multigene panels remain the most widely used clinical genetic test for ICC, including hypertrophic and dilated cardiomyopathy, LQTS, Brugada syndrome, ACM, FH, and hereditary aortopathies. These panels focus on genes with established disease associations, providing a practical balance between cost, turnaround time, and interpretive complexity [86]. Contemporary cardiac NGS panels achieve high read depth across exons and splice junctions and increasingly incorporate CNV calling for included genes [91].

For many ICC phenotypes, multigene panel testing is recommended as a first‐line genomic investigation when a clear inherited phenotype is suspected. According to the 2025 ESC Clinical Consensus Statement on integrating genetic testing into diagnostic pathways for cardiomyopathies, phenotype‐directed multigene panels remain the current first‐line genetic testing for cardiomyopathies, as they offer a practical balance between diagnostic yield and interpretive complexity. The diagnostic yield is highest in classic sarcomeric hypertrophic cardiomyopathy and slightly lower but still clinically useful in DCM, ACM, and LQTS [86, 92]. Positive results support clinical surveillance, lifestyle counseling (e.g., sports participation and occupational guidance), device therapy decisions, and cascade testing of relatives [90].

Key challenges include maintaining panel content alignment with evolving gene–disease evidence and avoiding inclusion of low‐evidence genes that may increase VUS burden [86]. Ancestry‐related bias in reference genomic databases contributes to higher VUS rates and reduced diagnostic certainty in many non‐European populations, highlighting the need for more diverse genomic resources [93]. Despite these limitations, targeted gene panels are expected to remain a cornerstone of clinical genomic cardiology in many healthcare systems in the foreseeable future [94].

### 4.3 Structural Variants (SVs) and Copy Number Variants

SVs and CNVs represent an important but historically under‐recognized genetic mechanism in inherited cardiomyopathies, arrhythmia syndromes, and congenital heart disease. Copy number variants affecting established ICC genes may cause haploinsufficiency or dosage effects comparable to single‐nucleotide variants. Large chromosomal CNVs also contribute to syndromic congenital heart disease and complex cardiac phenotypes [86].

Next‐generation sequencing has enabled in silico CNV calling from cardiac panels, WES, and especially WGS, increasing detection of submicroscopic deletions and duplications [95]. Adding CNV analysis to routine ICC sequencing provides a modest but clinically meaningful increase in diagnostic yield [86]. Interpretation of CNVs requires consideration of variant size, gene content, inheritance pattern, population frequency, and curated clinical evidence [96].

Emerging long‐read sequencing and hybrid short/long‐read strategies improve resolution of complex SVs, repeated regions, and large insertions or inversions that remain challenging for short‐read technologies [97]. Pilot cardiac genomic studies suggest that long‐read sequencing may uncover pathogenic SVs missed by conventional clinical pipelines, particularly in previously unresolved ICC cases [98]. As sequencing costs decrease, SV and CNV analysis is expected to be more systematically integrated into genomic cardiology workflows [85].

### 4.4 Polygenic Risk Scores and Integrated Risk Modeling

Polygenic risk scores quantify inherited susceptibility to complex cardiovascular diseases by aggregating the effects of multiple common variants. PRS derived from large GWAS and biobank studies can identify individuals at elevated lifelong risk who may not be detected using conventional clinical risk models alone [99, 100]. Although PRS performance varies across populations because discovery data sets differ by ancestry, ongoing multi‐ancestry GWAS efforts aim to improve calibration and clinical applicability [101, 102]. The 2025 AHA Policy Statement on Genetic and Genomic Testing emphasizes that although germline genotypes are static, genetic risk prediction remains inherently dynamic and requires consideration of many new policies. The statement notes that PRS continue to demonstrate increasingly predictive capabilities, providing notable opportunities to interrupt disease development early in the pathogenesis. However, the statement also highlights barriers to broad clinical implementation, including cost‐effectiveness, the implications of PRS for life and long‐term disability insurance, and the need for prospective trials to define the most efficacious PRS paradigms [103].

Current evidence supports using PRS as a risk‐enhancing marker rather than a standalone diagnostic tool. Integration of PRS with clinical risk factors improves prediction performance and supports risk reclassification, particularly in primary prevention settings [99]. The MI‐GENES randomized clinical trial provided the first prospective evidence that disclosure of a 10‐year coronary heart disease risk score that included a polygenic risk score to individuals at intermediate risk was associated with a lower incidence of major adverse cardiovascular events after a decade of follow‐up (hazard ratio 0.20, 95% CI: 0.04–0.94; *p* = 0.042), likely due to more frequent and prolonged statin use leading to lower LDL‐C levels [104]. However, this was a small single‐center trial and requires replication in larger, more diverse populations before definitive conclusions can be drawn. The 2025 AHA Policy Statement underscores the importance of strategic investments in biobanking and genomic research across all populations to enhance variant interpretation and improve risk prediction models [103]. Until such evidence emerges, PRS should be considered an adjunctive tool in selected scenarios rather than a basis for widespread clinical decision‐making.

Integrated clinical–genetic risk models extend PRS utility by incorporating monogenic variant information and traditional cardiovascular risk factors such as age, blood pressure, lipid levels, smoking status, and diabetes. Some approaches also integrate family history, coronary artery calcium imaging, and circulating biomarkers to improve stratification [4, 70, 105].

Pilot implementation studies suggest that individuals at high genetic risk may derive greater benefit from intensive preventive therapy, including high‐intensity statins and PCSK9 inhibition strategies [106]. However, prospective clinical trials are required to confirm outcome benefits, cost‐effectiveness, and equitable applicability across healthcare systems [107].

### 4.5 Pharmacogenomics

Pharmacogenomics enables personalization of cardiovascular drug therapy by incorporating genetic variants that influence drug metabolism, transport, and molecular targets. The strongest clinical evidence exists for statins, clopidogrel, and warfarin therapy [108]. Variants in *SLCO1B1* are associated with an increased risk of statin‐induced myopathy, particularly with simvastatin, and are incorporated into several pharmacogenomic dosing recommendations [109]. Although genotype‐guided statin selection may improve treatment adherence in high‐risk patients, routine population‐wide testing is not yet universally recommended [110].

Loss‐of‐function variants in *CYP2C19* reduce activation of clopidogrel and are associated with higher ischemic event rates following percutaneous coronary intervention (PCI) when standard dosing is used [111]. Genotype‐guided antiplatelet therapy using alternative agents such as prasugrel or ticagrelor in *CYP2C19* loss‐of‐function carriers is supported by clinical trial and registry data, particularly in high‐risk acute coronary syndrome (ACS) or PCI populations [112]. Warfarin dosing algorithms incorporating *CYP2C9* and *VKORC1* genotypes improve early anticoagulation control but have become less dominant with the widespread adoption of direct oral anticoagulants [113].

Practical implementation challenges include optimal timing of testing, reimbursement policies, clinician familiarity with genomic interpretation, and integration of pharmacogenomic results into electronic health record (EHR)–based decision support systems. Preemptive multigene pharmacogenomic panels that can be stored for lifetime clinical use are being evaluated in several healthcare systems [108]. Effective point‐of‐care decision support that translates genotype information into actionable prescribing guidance is essential for clinical adoption [114]. Despite strong evidence for specific pharmacogenomic applications, implementation barriers persist. These include limited clinician familiarity with genomic interpretation, lack of integration into clinical workflows, and uncertainty regarding cost‐effectiveness. Preemptive testing strategies are being evaluated, but prospective data demonstrating improved clinical outcomes from genotype‐guided prescribing remain limited.

RNA‐based and gene‐editing therapies represent an emerging frontier in cardiovascular therapeutics, but they must be clearly distinguished from current genomic risk assessment and clinical practice. Currently approved RNA‐targeted therapies include small interfering RNA (siRNA) agents such as inclisiran, which targets PCSK9 and is approved for lowering LDL cholesterol in high‐risk atherosclerotic CVD populations [105]. Additional RNA‐based approaches, including antisense oligonucleotides targeting ANGPTL3 and LPA, remain under clinical investigation. In contrast, gene‐editing technologies such as clustered regularly interspaced short palindromic repeat (CRISPR) remain predominantly at the preclinical stage.

As reviewed by Liu and Olson (2022), CRISPR studies in cardiomyopathy and arrhythmia models have shown potential for correcting or silencing pathogenic variants in sarcomere and ion‐channel genes. However, substantial barriers remain before these approaches can be translated to clinical practice [115]. Vo (2025) reported that while CRISPR‐Cas9 achieved high editing efficiencies (25%–85%) in animal models using AAV vectors, safety concerns, including off‐target effects, immune responses to viral vectors, and AAV vector integration at cut sites, remain unresolved [116]. Musolino and colleagues further emphasized that developing cardiovascular gene therapeutics represents a substantial technical challenge, particularly in identifying and developing effective delivery systems with acceptable safety profiles. Ethical, regulatory, and access issues are particularly salient for high‐cost gene editing approaches [117]. As Khera and colleagues emphasize, gene editing and gene therapy require strict ethical, safety, and regulatory oversight. These technologies should not be conflated with established genomic risk assessment tools. They are presented here as a therapeutic frontier rather than a component of current precision cardiovascular care [118].

### 4.6 Functional Genomics and Experimental Models

Functional genomics links genetic variation to molecular and cellular mechanisms, helping to interpret variants and identify therapeutic targets [119]. Multiomics technologies, including transcriptomics, epigenomics, and single‐cell omics, map disease‐relevant regulatory networks in cardiac and vascular tissues. Integrative GWAS analyses have helped nominate causal genes at susceptibility loci for CAD and related cardiovascular traits, including effects on vascular cell biology [120]. Induced pluripotent stem cell (iPSC)–derived cardiomyocytes combined with CRISPR editing provide patient‐specific and isogenic models for studying arrhythmia syndromes and cardiomyopathies. These models are useful for assessing variant pathogenicity, drug screening, and phenotypic rescue experiments. Animal models such as mouse and zebrafish systems remain important for in vivo functional validation of cardiovascular genes and pathways [119].

High‐throughput assays such as multiplexed variant effect mapping are emerging for key cardiac genes, enabling parallel functional assessment of many variants. These data sets feed into variant classification frameworks and can reduce the VUS burden for clinically important loci [121]. Combined with multiomics network approaches, Functional genomics bridges genetic associations to mechanistic understanding. However, translation to patient care requires scalable functional assays and improved variant interpretation frameworks [122].

### 4.7 Clinical Applications of Genomic Cardiology: A Framework Based on Clinical Readiness

Genomic cardiology is progressively transitioning from specialized centers into broader clinical practice, increasingly informing risk assessment, diagnosis, prognosis, and therapeutic decision‐making across the CVD spectrum [123]. Implementation is currently more common in high‐resource settings and ICC programs, with multiomics data and digital health tools accelerating translation toward early prevention and routine clinical decision‐making [88]. Consistent with the three‐tier framework introduced in the Introduction, this section organizes the clinical applications of genomic cardiology according to their level of evidence, guideline support, and readiness for routine practice. Tier 1 includes applications that are already part of the standard of care in guideline‐endorsed contexts. Tier 2 covers tools that have demonstrated promise and may be considered in selected patients, but for whom broader implementation requires additional prospective validation. Tier 3 encompasses emerging technologies that remain predominantly experimental and are not yet appropriate for routine clinical decision‐making. A summary of the evidence hierarchy and clinical readiness for these applications is provided in Table 3, while a typical workflow for integrating these approaches into clinical practice is illustrated in Figure 2.

**Table 3:** Evidence hierarchy and clinical readiness of genomic applications in cardiovascular medicine.

Clinical application	Target population	Evidence level	Guideline position	Clinical utility	Principal limitations	Tier
Targeted multigene panels for HCM, DCM, LQTS, Brugada, FH, aortopathies	Patients with suspected inherited cardiac conditions	High (multiple guidelines)	ESC/AHA Class I recommendations	Diagnosis, cascade screening, device decisions, lifestyle counseling	VUS burden, ancestry bias, low evidence genes	1
Cascade testing for ICC and FH	At‐risk relatives of pathogenic variant carriers	High	Strong guideline support	Early identification, preemptive prevention, family surveillance	Equitable access, counseling infrastructure, psychosocial impact	1
*CYP2C19* genotyping for clopidogrel	PCI/ACS patients	Moderate high	CPIC/ESC guideline	Individualized antiplatelet therapy	Timing of testing, reimbursement, clinician education	1
*SLCO1B1* genotyping for statins	Patients initiating statin therapy	Moderate	CPIC guideline	Myopathy risk reduction, adherence improvement	Limited routine adoption, cost‐effectiveness	1
PRS for CAD	Primary prevention patients	Moderate (observational)	ACC/AHA as risk‐enhancing factor (not standalone)	Risk reclassification, earlier statin initiation	Ancestry bias, limited prospective outcome data	2
WES/WGS after negative targeted panel	Selected ICC patients with high clinical suspicion	Moderate	Selective use, no universal guideline	Diagnosis in heterogeneous/atypical phenotypes	High VUS rate, cost, interpretation complexity	2
PRS for other CVDs (AF, stroke, HF)	Selected patients	Low moderate	No specific guideline	Exploratory risk stratification	Limited validation, ancestry bias	2
AI‐optimized integrated risk models	Research settings	Low (proof of concept)	No guideline support	Exploratory risk prediction	Reproducibility, bias, external validation needed	3
Multi‐omics integration (transcriptomics, proteomics, metabolomics)	Research settings	Very low	No guideline support	Mechanistic insights, biomarker discovery	Cost, standardization, clinical validation	3
CRISPR gene editing/RNA therapies	Preclinical/early phase trials	Very low	No guideline support	Potential curative or durable therapy	Off‐target effects, safety, cost, access	3
Digital twins	Experimental	Very low	No guideline support	In silico therapy testing, dynamic risk forecasting	Validation, governance, data integration	3

**Figure 2:**
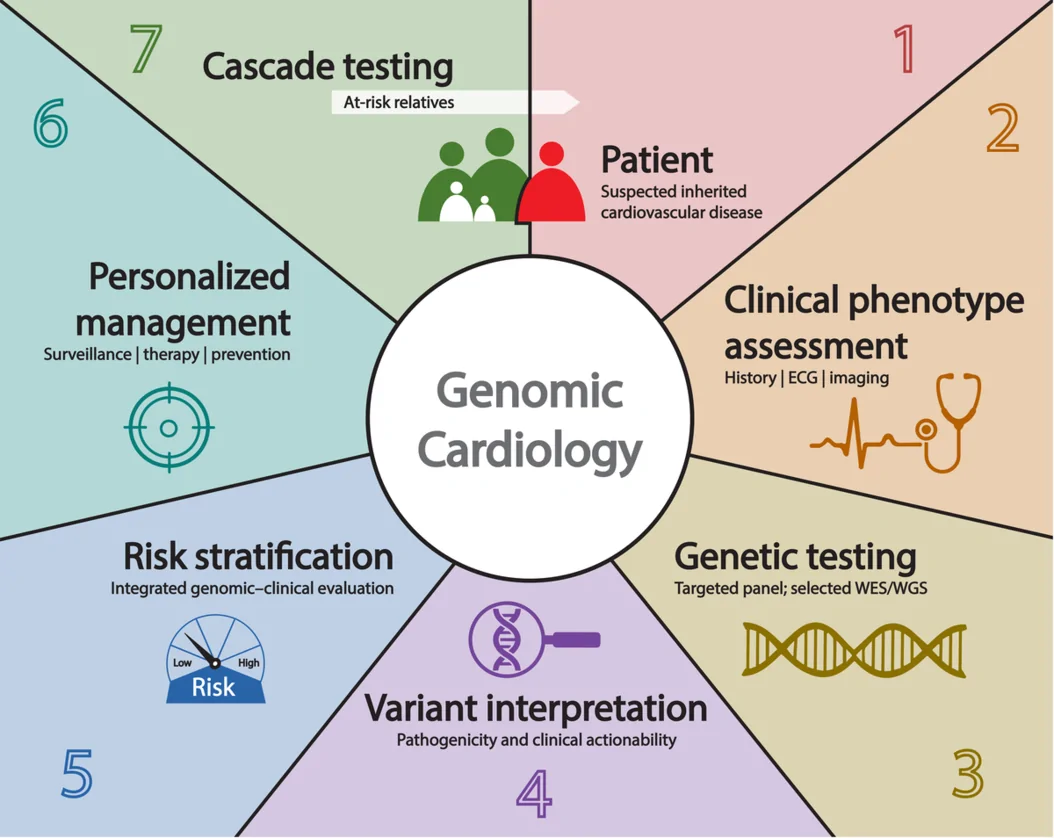
Clinical workflow of genomic cardiology. Schematic overview of a clinical pathway for patients with suspected inherited cardiovascular disease. The workflow starts from (1) a patient with suspected inherited cardiovascular disease, followed by (2) clinical phenotype assessment, (3) selection of appropriate genetic testing, (4) variant interpretation with attention to pathogenicity and clinical actionability, (5) integrated genomic–clinical risk stratification, and (6) personalized management including surveillance, therapy, and prevention. The pathway culminates in (7) cascade testing of at‐risk relatives to enable risk assessment and prevention.

### 4.8 Tier 1—Guideline‐Supported Genomic Applications in Routine Practice

Tier 1 encompasses genomic applications with strong professional guideline endorsement and established integration into routine cardiovascular care. Genetic testing is considered standard of care in many inherited cardiac conditions, including hypertrophic and dilated cardiomyopathy, LQTS and Brugada syndromes, ACM, and familial aortopathies [89]. Multigene panels remain the first‑line genomic investigation for these phenotypes, providing a practical balance between cost, turnaround time, and interpretive complexity. According to the 2025 ESC Clinical Consensus Statement, panels should include all ClinGen‐validated cardiomyopathy genes [92]. Importantly, expanding panels beyond this core set adds little diagnostic yield but significantly increases the VUS burden. Positive results directly support clinical surveillance, lifestyle and occupational counseling, decisions on device therapy, and cascade testing of at‑risk relatives.

Cascade testing represents one of the most powerful and cost‑effective applications of genomic cardiology. Once an index patient has a pathogenic or likely pathogenic variant identified, targeted testing of relatives enables early identification of carriers who can enter surveillance and preventive programs before symptoms develop. Population‑based or health system‑wide FH and ICC screening initiatives demonstrate that combining genomic cascade testing with registry infrastructure can substantially increase detection rates [124].

Pharmacogenomic markers with strong guideline support also fall within Tier 1. Variants in *CYP2C19* influence clopidogrel metabolism, and genotype‑guided antiplatelet therapy using alternative agents such as prasugrel or ticagrelor is supported by clinical trial data in high‑risk ACS or PCI populations [125]. Similarly, *SLCO1B1* genotyping informs statin‐associated myopathy risk and supports individualized prescribing decisions. Integration of these pharmacogenomic results into EHRs through clinical decision support (CDS) tools is increasingly feasible and facilitates consistent, guideline‐aligned care [126].

### 4.9 Tier 2—Emerging Applications With Selective Clinical Utility

Tier 2 includes approaches with encouraging preliminary data and potential utility in specific patient subgroups, but they still require further prospective validation before broad clinical deployment. PRS for CAD consistently stratify lifetime risk and may improve risk reclassification among patients in borderline clinical categories [127]. Current professional guidance, including the 2025 AHA Policy Statement, positions PRS as a risk‐enhancing factor rather than a standalone diagnostic test [103].

They may be considered in selected primary prevention scenarios where additional risk information could influence management decisions, such as earlier statin initiation or more intensive lipid‐lowering therapy [128]. However, several important caveats remain. First, although the MI‐GENES 10‐year follow‐up trial showed promising results [104], this was a small single‐center study and requires replication in larger, more diverse populations. Second, most GWAS participants have European ancestry, and polygenic scores derived from European populations often perform poorly in non‐European populations [129]. Lupi et al. showed that relative accuracy varies substantially across the genome, and even for ancestries with low overall relative accuracy (e.g., African), some genomic regions remain highly portable. Third, the 2025 AHA Policy Statement emphasizes the need for prospective trials to define the most efficacious PRS paradigms, as well as infrastructure investment and field consistency in genomic data management, iterative analysis, and clearly defined criteria for re‐reporting findings [103]. Until prospective validation and cost‐effectiveness data become available, PRS should be used judiciously and primarily as an adjunct to, rather than a replacement for, established clinical risk assessment tools.

In monogenic lipid disorders such as FH, genomic diagnosis enables early initiation of intensive lipid‐lowering therapy and identification of candidates for emerging RNA‐ or gene‐targeted treatments [124]. Family‐based genomic screening identifies asymptomatic high‐risk relatives before clinical onset, supporting pre‐emptive prevention strategies.

WES and WGS may be considered after negative targeted panel testing in patients with strong clinical suspicion of an ICC, particularly in phenotypes with high genetic heterogeneity or atypical presentations. Diagnostic yield varies across phenotypes and is generally higher in hypertrophic cardiomyopathy than in heterogeneous dilated or nonischemic cardiomyopathies. Interpretation of WES/WGS results requires multidisciplinary expertise involving cardiology, clinical genetics, genetic counseling, and bioinformatics [88].

Genomic data also complement conventional risk scores by identifying individuals with elevated inherited susceptibility who may otherwise be categorized as low or intermediate risk based on traditional factors alone [107]. Integrated genetic risk models that combine PRS with monogenic variant information and traditional cardiovascular risk factors are being evaluated in research settings and may eventually support shared decision‐making, although prospective outcome validation remains a critical gap.

### 4.10 Tier 3—Investigational and Experimental Approaches

Tier 3 covers emerging technologies that remain predominantly experimental and are not yet ready to influence routine clinical management. AI‐optimized models that integrate PRS with biomarkers and imaging features demonstrate improved discrimination compared with genetics‐only models in proof‐of‐concept studies, but prospective outcome validation and external calibration across diverse populations remain limited [130]. Similarly, AI‐driven approaches are being tested to automate variant interpretation triage and to combine genomic findings with clinical data for risk stratification [131]. Key challenges, including algorithmic bias, data quality, reproducibility, interpretability, and workflow integration, must be addressed before these tools can be adopted in routine practice.

Multi‑omic signatures, including transcriptomic, proteomic, metabolomic, and epigenomic markers, are emerging as prognostic tools in HF and pulmonary hypertension, although their clinical use remains early and largely confined to research settings [123]. Network‑based integration of these molecular layers has identified regulatory modules implicated in cardiometabolic disease and arrhythmias, but prospective evidence and cost‑effectiveness analyses do not yet support routine clinical adoption.

Gene editing and RNA‐based therapies are explicitly investigational and should not be conflated with established genomic risk assessment tools. Currently approved RNA‐targeted therapies include inclisiran (siRNA targeting PCSK9) for lipid lowering in high‐risk atherosclerotic cardiovascular disease. Other RNA‐based agents, such as antisense oligonucleotides targeting ANGPTL3 and LPA, remain under clinical investigation. Clustered Regularly Interspaced Short Palindromic Repeats gene‐editing technologies remain predominantly preclinical, with substantial barriers including off‐target effects, immune responses, long‐term safety, manufacturing complexity, high costs, and equitable access remaining unresolved [116, 117]. Jurado Vélez and colleagues further emphasize that addressing these challenges is essential to ensure that the benefits of emerging genomic technologies, including gene therapies, are equitably distributed across diverse populations [123]. These technologies are presented here as an emerging therapeutic frontier rather than a component of current precision cardiovascular care. Similarly, digital twin computational models that integrate genomic, imaging, physiological, and environmental data to simulate individual cardiovascular trajectories remain largely experimental and require extensive validation before any clinical application can be considered.

### 4.11 Practical Guidance for Test Selection and Result Interpretation in Inherited Cardiac Conditions

For the practicing cardiologist, several key clinical questions arise when considering genetic testing in patients with suspected inherited cardiac conditions. The following evidence‐based guidance addresses these questions.

Who should be tested? According to the 2018 HFSA/ACMG guideline, consider genetic testing in patients with clinical suspicion of an inherited cardiac condition, including hypertrophic or dilated cardiomyopathy, LQTS or Brugada syndromes, ACM, FH, or hereditary aortopathies. Testing is also indicated in families with unexplained sudden cardiac death and in patients with syndromic features suggestive of a genetic disorder [86, 89]. The 2025 ESC Clinical Consensus Statement emphasizes that cardiologists should systematically collect family history and detailed clinical phenotypes to support pre‐test hypotheses and guide testing strategies [92]. However, real‐world data from Longoni et al. demonstrate that genetic testing is profoundly underutilized across multiple inherited cardiovascular conditions, with only 0.8% of DCM patients, 1.6% of HCM patients, 1.2% of LQTS patients, and 1.5% of FH patients receiving genetic testing despite guideline recommendations [90].

Which test should be selected? For most well‐characterized inherited cardiac phenotypes, a targeted multigene panel is recommended as the first‐line genomic investigation. According to the 2025 ESC Clinical Consensus Statement, phenotype‐directed multigene panels remain the current first‐line genetic testing for cardiomyopathies, as they offer a practical balance between diagnostic yield and interpretive complexity [92]. Loong and colleagues similarly note that panels should include all ClinGen‐validated genes and that increasing the number of genes beyond this core set adds little diagnostic yield while substantially increasing VUS burden [86]. The diagnostic yield is highest in classic sarcomeric HCM (30%–60%) and lower but still clinically useful in DCM (10%–40%), ARVC (10%–50%), and LQTS (50%–75%) [88, 89].

When is a targeted panel preferable to WES or WGS? The 2025 ESC Clinical Consensus Statement notes that WES and WGS are not currently recommended as first‐line tests in adult patients with cardiomyopathies, because their diagnostic yield is similar to multigene panels, while they generate significantly more VUS and require management of secondary findings [92]. Loong and colleagues indicate that WES or WGS should be reserved for patients with negative panel testing but strong clinical suspicion, atypical or overlapping phenotypes, high genetic heterogeneity, or syndromic features that suggest involvement of multiple organ systems. WGS also detects non‐coding variants, structural rearrangements, and mitochondrial variants that may be missed by panels or exomes [86, 88].

How should a VUS affect management? The 2018 HFSA/ACMG guideline states that a variant of uncertain significance should not be treated as equivalent to a pathogenic or likely pathogenic variant. In isolation, a VUS should not drive irreversible clinical decisions (e.g., implantable cardioverter‐defibrillator implantation, transplant listing) or predictive cascade testing in relatives [89]. The 2025 ESC Clinical Consensus Statement emphasizes that VUS should be interpreted using a combination of computational tools, family segregation studies, and, when feasible, functional assays (RNA‐ or protein‐based), and underscores that many VUS classified in early literature are now being reclassified [92]. Papadopoulou et al. note that the majority of VUS are eventually downgraded to benign or likely benign, and clinicians should be extremely cautious with VUS management, as the erroneous use of such variants as pathogenic could have detrimental effects not only for the proband but also for relatives [88]. The 2025 AHA Policy Statement emphasizes that VUS should be re‐evaluated periodically as new evidence emerges, and patients should be counseled regarding the potential for reclassification over time [103].

When should relatives undergo cascade testing? The 2018 HFSA/ACMG guideline recommends that cascade testing should be offered to all at‐risk relatives immediately upon identification of a pathogenic or likely pathogenic variant in an index patient [89]. Knowles et al. demonstrate that cascade testing for FH is highly effective and cost‐effective, with an incremental cost‐effectiveness ratio of $2500 to $4500 per quality‐adjusted life‐year gained [124]. Loong and colleagues note that cascade testing begins with immediate family members, followed by second‐ and third‐degree relatives, until all at‐risk family members are identified and tested to the extent possible [86]. Targeted testing of the specific familial variant is cost‐effective and enables early identification of carriers who can enter surveillance and preventive programs before symptoms develop.

What findings should not alter treatment? The 2018 HFSA/ACMG guideline states that VUS, variants in low‐evidence genes, and variants that are not consistent with the patient's phenotype should not guide clinical management. Similarly, secondary or incidental findings in genes without established clinical actionability should be reviewed by a multidisciplinary team before any therapeutic decisions are made [89]. The 2025 ESC Clinical Consensus Statement reinforces that clinical decision‐making begins and ends with the clinical phenotype, and that genetic testing can be harmful when discrepancies between phenotype and genotype are ignored or overlooked [92]. All test selection and interpretation should be coordinated through multidisciplinary inherited cardiac condition teams involving cardiologists, clinical geneticists, genetic counselors, and bioinformatics specialists [86, 90].

## 5 Integration With Other‐Omics and Digital Health

Integration of genomics with complementary molecular and digital data layers extends precision cardiology beyond static DNA‐based risk toward dynamic characterization of disease biology [122]. Multiomics approaches, including transcriptomics, proteomics, metabolomics, and epigenomics combined with imaging, electrocardiographic (ECG) signals, and wearable‐derived metrics, enable more mechanistic models of cardiovascular risk, progression, and therapeutic response (Țapoș and colleagues). Figure 3 summarizes this multimodal integration framework [132].

**Figure 3:**
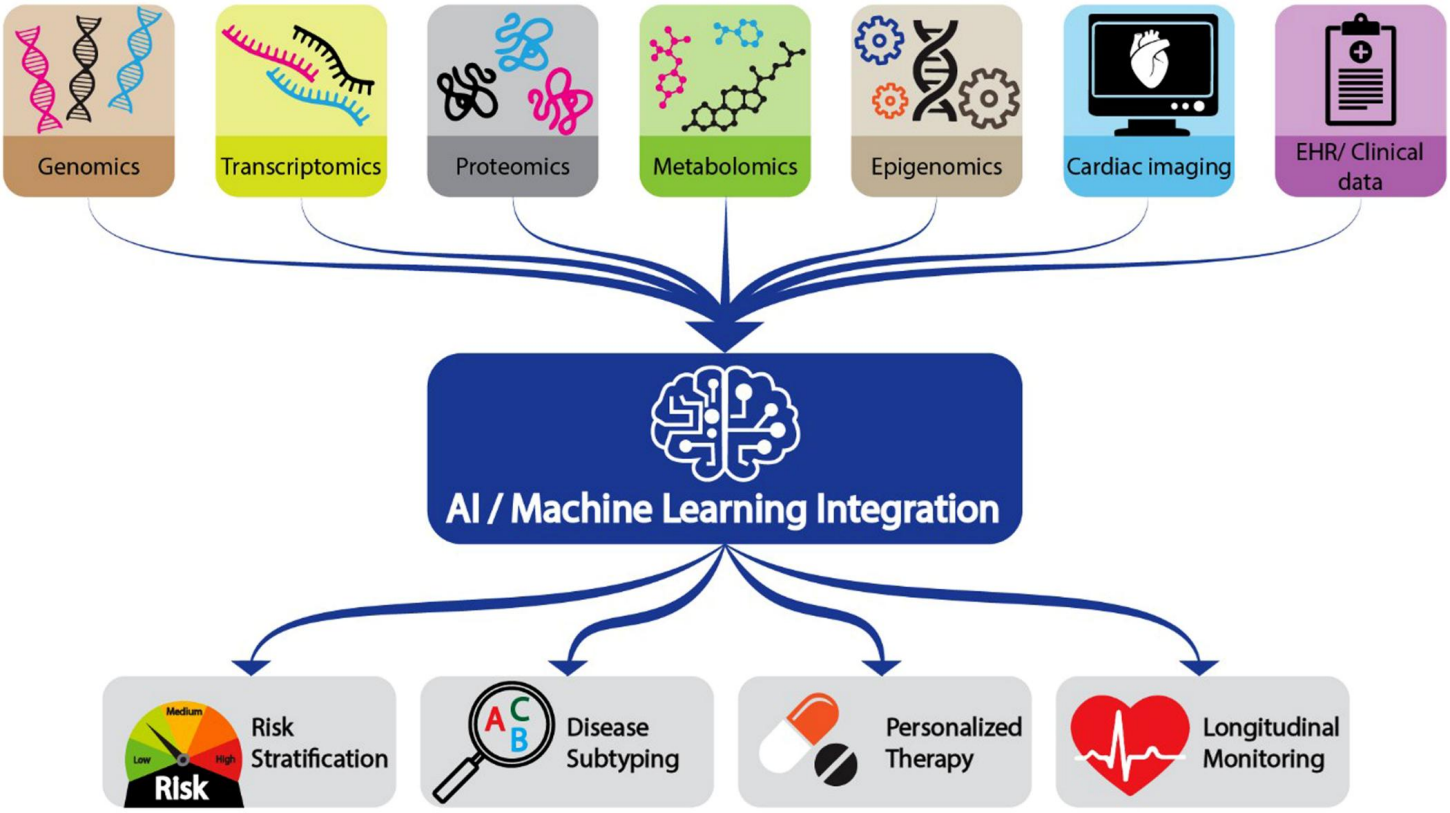
Multiomics and AI integration framework in precision cardiology. Schematic representation of the integration of genomics, transcriptomics, proteomics, metabolomics, epigenomics, imaging‐derived phenotypes, and digital health data through artificial intelligence–driven modeling. The framework shows how multimodal biological and clinical data translate into improved cardiovascular risk prediction, disease subtyping, therapy selection, and longitudinal monitoring.

### 5.1 Transcriptomics, Proteomics, Metabolomics, Epigenomics

Transcriptomic profiling has identified gene expression signatures associated with HF and arrhythmogenic substrates [133]. Circulating RNA species are being evaluated as biomarkers. Epigenomic analyses provide insight into regulatory mechanisms linking environmental exposures with inherited susceptibility [134]. Proteomic and metabolomic platforms offer functional readouts of pathway activation. However, routine clinical adoption of multi‐omic biomarkers remains limited, with most data derived from discovery‐phase studies rather than prospective validation [122]. According to Lin and colleagues, the integration of multiomics with machine learning (ML) has shown promising outcomes in cardiovascular studies, but the volume and complexity of data pose significant challenges for accurate description, utilization, and translation of biomedical data into clinical practice [135].

### 5.2 Imaging Omics and Electrocardiographic‐Based Phenotyping

Imaging omics uses machine learning to extract high‐dimensional phenotypes from echocardiography, cardiac MRI, and CT [136]. Correlating imaging features with genomic data enables identification of mechanistic endotypes. Advanced ECG analytics use deep learning to extract latent features associated with structural heart disease and arrhythmia [136]. Proof‑of‑concept studies suggest potential for enhanced prediction, but external validation and calibration across populations are still required.

### 5.3 Artificial Intelligence and Multimodal Integration

Artificial intelligence and machine learning provide frameworks for integrating genomic, multiomic, imaging, ECG, and clinical data [136]. Machine learning approaches have been used to refine PRS construction [101]. Multimodal AI models have shown improved discrimination in experimental settings [136]. However, as highlighted by Quer and colleagues, AI algorithms would need to be prospectively assessed for their real‐world effectiveness and developed within a robust regulatory framework with a focus on high‐value and equitable care [118]. Lin and colleagues further note that most AI and multi‐omics models in cardiovascular research remain at the proof‐of‐concept stage, with limited external validation and translation to clinical practice [135].

Specific challenges include interpretability, reproducibility, ancestry‑related bias when trained on homogeneous data sets, data quality, and workflow integration [137]. Khera, Oikonomou and colleagues emphasize that data privacy and security, interoperability across health systems, and appropriate regulatory controls are essential safeguards for realizing an AI‐enabled future [118].

Professional societies increasingly emphasize transparent reporting, external validation, and ongoing monitoring for unintended disparities [138]. Development of explainable AI and recalibration strategies across diverse populations remains central to trustworthy implementation [136]. Critically, most AI and multimodal models remain at the proof‑of‑concept stage; none have been prospectively validated or shown to improve cardiovascular outcomes in routine clinical practice.

### 5.4 Wearables and Digital Phenotyping

Wearable devices generate continuous physiological data that, when integrated with genomic information, may clarify how inherited risk manifests dynamically [139].

Digital phenotyping enables monitoring of therapeutic responses in individuals with elevated genetic risk. However, disparities in access to digital technologies and concerns regarding surveillance, data security, and consent must be addressed to ensure equitable deployment [122]. Khera, Oikonomou et al. note that the selectivity among those who opt to use wearable devices likely exerts a potent confounding effect, and the benefits of digital biomarkers must be carefully evaluated in diverse populations [118].

### 5.5 EHR Integration and Learning Health Systems

EHR integration provides the infrastructure linking genomics, multi‐omics, imaging, and digital health data to clinical workflows [126]. Learning health system models iteratively feed data into analytic pipelines to recalibrate risk algorithms [140].

Early implementation programs demonstrate that EHR‑embedded genomic tools can be triggered in relevant clinical contexts [130]. However, interoperability standards, privacy safeguards, and equitable infrastructure development will determine whether these systems reduce or exacerbate disparities [141]. AI and multiomics integration remain experimental. Prospective validation, algorithmic fairness, and regulatory oversight are essential before clinical adoption [118].

## 6 Challenges and Limitations

Genomic cardiology operates at the intersection of rapidly advancing molecular technologies and persistent structural, clinical, and societal constraints that shape who benefits from precision approaches and how safely they are implemented [130]. Addressing these limitations is essential to ensure that precision cardiology evolves as a sustainable and equitable clinical framework rather than a collection of isolated proof‐of‐concept applications [123].

### 6.1 Population Bias and Ancestry Disparities

Most cardiovascular GWAS and PRS development cohorts still overrepresent individuals of European ancestry, with African, Indigenous, and many Asian and Latin American populations markedly underrepresented [93]. This imbalance limits the generalizability of variant effect estimates, gene–disease associations, and PRS performance across global populations [123]. Transferability studies consistently demonstrate reduced PRS discrimination when models derived from European cohorts are applied to African ancestry populations, even within large biobanks [123].

Differences in allele frequencies, linkage disequilibrium patterns, and environmental exposures further complicate cross‐ancestry prediction [93]. Without deliberate diversification of discovery and validation data sets, genomic cardiology risks exacerbating existing cardiovascular health inequities [123]. Large multi‐ancestry GWAS, ancestry‐specific calibration, and intentional recruitment of underrepresented populations are therefore central to responsible implementation [93].

### 6.2 Interpretability and Clinical Utility of PRS

Although PRS can stratify relative cardiovascular risk, their absolute predictive value and clinical actionability remain debated. Many scores explain meaningful variation in susceptibility but do not yet meet standards expected of standalone screening tools in population health contexts. Clinicians frequently report uncertainty about communicating probabilistic risk and translating it into management decisions beyond established guideline thresholds. Evidence that PRS‐guided interventions improve hard cardiovascular outcomes remains limited, with most data focused on risk reclassification or prescribing changes. Overstatement of clinical utility may undermine confidence if benefits prove modest or uneven across populations [142]. Clear implementation frameworks are needed to define when PRS function as risk enhancers, when they inform treatment thresholds, and when they remain investigational tools (Figure 3). The 2025 AHA Policy Statement underscores that despite promising capabilities, PRS face substantial barriers to broad clinical implementation, including uncertain cost‐effectiveness, implications for insurability, and a lack of prospective trial data to define optimal usage paradigms [103]. While the MI‐GENES trial provided a single small‐scale signal of potential benefit, large‐scale replication is lacking [104]. Moreover, portability across diverse ancestries remains a major concern, as relative accuracy varies substantially along the genome and is lowest in populations underrepresented in discovery GWAS [129]. Overstatement of clinical utility may undermine confidence if benefits prove modest or uneven across populations [130].

### 6.3 Variant Interpretation and Variant of Uncertain Significance Burden

Variant interpretation remains a major bottleneck in genomic cardiology, with many rare variants classified as VUS, particularly in underrepresented populations. Limited functional data, incomplete ancestry‐matched reference databases, and evolving gene–disease evidence contribute to uncertainty. Both false reassurance and inappropriate escalation carry significant consequences in high‐risk cardiac conditions. Guidelines such as the ACMG/AMP framework provide structure, but their application varies, and many laboratories lack systematic reanalysis pipelines [123].

From a clinical perspective, a variant of uncertain significance should not be treated as equivalent to a pathogenic or likely pathogenic variant. According to the 2018 HFSA/ACMG guideline, a VUS should not, in isolation, drive irreversible clinical decisions (e.g., implantable cardioverter‑defibrillator implantation, transplant listing) or predictive cascade testing in relatives [89]. The 2025 ESC Clinical Consensus Statement reinforces that VUS should be interpreted using a combination of computational tools, family segregation studies, and, when feasible, functional assays (RNA‑ or protein‑based), and underscores that many VUS classified in early literature are now being reclassified [92]. Papadopoulou et al. demonstrated that the majority of VUS are eventually downgraded to benign or likely benign, and clinicians should be extremely cautious with VUS management, as the erroneous use of such variants as pathogenic could have detrimental effects not only for the proband but also for relatives [88].

The 2025 AHA Policy Statement emphasizes that VUS should be re‑evaluated periodically as new evidence emerges, and patients should be counseled regarding the potential for reclassification over time [103]. High‐throughput functional assays and multiplexed variant effect mapping are beginning to reduce uncertainty for selected genes, though coverage remains incomplete [122]. Formal reclassification and patient recontact strategies are needed to balance evolving knowledge with clinical stability [123]. The section “Practical Guidance for Test Selection and Result Interpretation in Inherited Cardiac Conditions” discusses clinical decision‐making regarding VUS and provides an actionable framework for clinicians.

### 6.4 Cost‐Effectiveness and Accessibility

Comprehensive genomic testing and multiomics integration remain expensive and unevenly available, with access often tied to academic centers, private insurance, or research participation. While sequencing costs per base have dropped, downstream expenses for interpretation, counseling, infrastructure, and follow‐up still constrain widespread implementation. Many cost‐effectiveness analyses show favorable ratios for targeted ICC testing and cascade screening, but less clear value for population‐wide sequencing or PRS testing. Reimbursement policies vary widely across jurisdictions and often lag behind evidence, creating uncertainty for both providers and patients [143].

Digital divides and structural barriers mean that communities already experiencing high cardiovascular burdens may have the least access to precision tools [141]. Sustainable precision cardiology models must integrate targeted genomic strategies with public health infrastructure and community engagement [123].

### 6.5 Health System Integration and Workforce Constraints

Successful integration of genomics into routine cardiovascular care requires system‐level transformation. Many clinics lack standardized referral pathways, embedded genetic counseling support, and clinician training in genomic interpretation. Fragmentation across cardiology, genetics, primary care, and laboratory services can result in inconsistent follow‐up and duplication [130].

EHRs are often not optimized for structured genomic data storage or automated clinical decision support. Scaling precision cardiology will require dedicated implementation teams, institutional leadership, and workflow designs aligned with local practice realities [143].

### 6.6 Data Privacy, Governance, and Security

Genomic and multiomic data are uniquely identifying and highly sensitive, raising concerns about privacy, data misuse, and long‐term security [144]. Cardiovascular genomic data sets are increasingly linked with EHRs, imaging archives, and digital health streams, expanding both their scientific value and their risk profile [145]. Patients express worries about discrimination, unauthorized access by employers or insurers, and loss of control over data as it is shared across institutions and borders [146]. Robust governance frameworks, including transparent consent processes, clear data‐sharing agreements, and independent oversight, are essential to maintain trust [147]. Technical safeguards such as encryption, access controls, and audit trails must be matched by cultural practices that prioritize privacy and respect for participants [137]. Ongoing dialog with patients and communities is critical as AI and large‐scale analytics increase the potential to infer sensitive information from ostensibly de‐identified cardiovascular data sets [148].

### 6.7 Ethical, Legal, and Social Implications (ELSI)

Ethical, legal, and social questions around cardiovascular genomics mirror broader debates about genetic risk prediction but are sharpened by the high stakes of cardiac outcomes [149]. Key concerns include fair access to testing and therapies, potential stigmatization based on genetic risk, and responsible communication of probabilistic information [148]. There is also debate about how much responsibility clinicians have to recontact patients as variant interpretations evolve and how to manage incidental and secondary findings [150]. Legal frameworks for genetic nondiscrimination, consent, and data protection vary across countries and may not fully address emerging uses of PRS, AI‐driven models, or digital phenotyping [151]. Ensuring that the benefits and burdens of precision cardiology are shared fairly requires intentional focus on justice, community engagement, and inclusion of patient voices in governance [152]. Ethical guidance is gradually adapting from monogenic rare disease contexts to the more diffuse, probabilistic nature of polygenic cardiovascular risk [148].

### 6.8 Evolving Guidelines and Implementation Science

Clinical guidelines for genomic testing in ICC are well established, whereas recommendations for PRS, multiomics integration, and AI‐enabled tools are still developing [153]. Most guidelines endorse targeted testing in well‐defined ICC phenotypes and cascade screening but urge caution around population‐wide genomic screening [154]. For PRS, major societies currently position scores as risk‐enhancing factors that may inform decisions in selected scenarios rather than as standalone tests [155]. Implementation frameworks drawn from implementation science emphasize staged rollout, local adaptation, and continuous evaluation [156]. Learning health systems and regional genomic networks are testing models that embed precision cardiology tools in real‐world workflows while monitoring equity and outcomes [157]. Clear guidance on training, infrastructure, and minimum data set standards will be essential for broader adoption [158].

### 6.9 Multiancestry PRS and Large Biobanks/Consortia

Large, diverse biobanks are essential to improving fairness in genomic risk prediction [159]. Multi‐ancestry PRS frameworks that incorporate ancestry‐aware weighting and recalibration demonstrate improved performance compared with earlier single‐ancestry models [101]. Biobanks that link genomics with deep phenotyping, such as imaging and multi‐omics, provide fertile ground for discovering ancestry‐specific biology and refining models [160]. Yet, even the largest current resources still underrepresent many regions and communities, and sustained investment is needed to build and maintain truly global biobanks [161]. Community partnerships and benefit‐sharing arrangements are critical to avoid extractive research practices [162]. Methodological work on transfer learning, federated learning, and privacy‐preserving analytics may accelerate progress while respecting local governance [163].

### 6.10 Next‐Generation Gene/RNA Therapies and Digital Twins

Next‐generation gene and RNA therapies promise one‐time or highly durable interventions on causal pathways but raise questions about safety, cost, and long‐term monitoring [164]. Off‐target editing, immune responses to vectors, and uncertainties about lifetime effects are key scientific and regulatory concerns [165]. High upfront costs and complex manufacturing also pose major challenges for equitable access [151]. Digital twins (computational models that integrate genomic, imaging, physiological, and environmental data to simulate individual cardiovascular trajectories) are emerging as a conceptual and technical frontier. These systems could eventually allow in silico testing of therapies and dynamic risk forecasting but currently remain largely experimental [166]. Ensuring transparency, validation, and governance for digital twins that incorporate highly sensitive genomic and multiomic data will be a major challenge as the field advances [167].

## 7 Future Directions

Future research in genomic cardiology should prioritize increasing ancestral diversity in biobanks, as the predominance of European participants continues to limit the portability and fairness of PRS. Recalibration and ancestry‐aware optimization of PRS for target populations are essential to ensure equitable risk prediction. Early‐life application, particularly in individuals under 40 years of age, may maximize preventive benefit, while additional data are needed in older adults (> 80 years) and in prospective interventional studies to validate observational findings. Improving phenotypic specificity, especially for HF and cardiomyopathies, and integrating PRS with established clinical risk models may enhance prevention strategies, enable earlier intervention, and support more informed healthcare decision‐making [45].

In DCM, expanding GWAS and polygenic analyses across diverse populations remains critical. Integrating polygenic susceptibility with rare pathogenic variant data and clinical risk models may improve the prediction of disease onset, penetrance, and severity. Validation in real‐world clinical settings, including asymptomatic variant carriers and family members, is required to establish actionable risk thresholds and demonstrate clinical utility. Approaches that combine monogenic and polygenic risk, incorporate multitrait GWAS, and leverage imaging‐derived phenotypes may further refine individualized prevention and management strategies. International collaboration and well‐characterized, deeply phenotyped cohorts will be essential to achieve these goals [168].

Beyond single‐layer genetic models, multi‐ancestry PRS frameworks that integrate data from diverse GWAS cohorts and apply ancestry‐informed weighting show improved performance compared with earlier single‐ancestry scores. Continued validation in multiethnic populations, refinement through integration with genomic, biomarker, lifestyle, and imaging data, and standardized implementation workflows are key next steps [169].

Single‐cell and multiomics technologies are providing deeper mechanistic insights into CVD by resolving cell‐type–specific transcriptomic profiles, spatial organization, and intercellular communication networks. These approaches enable identification of disease‐specific regulatory pathways and may support development of more precise, patient‐specific therapeutic strategies [12].

## 8 Conclusion

Genomic cardiology has already transformed the management of selected inherited CVDs by enabling precise genetic diagnosis, risk stratification, and family‐based cascade screening. However, its impact remains more limited and largely prospective for common polygenic conditions, where the clinical utility of PRSs, broad population screening, multiomics, AI‐supported models, and emerging therapies requires further validation. The field integrates information from rare high‐impact variants and polygenic susceptibility, together with molecular, imaging, and digital health data, to support personalized cardiovascular care, but the extent of clinical translation varies substantially across applications.

Despite rapid technological progress, several barriers limit widespread clinical implementation. These include ancestry‐related bias in genomic data sets, uncertainty in variant interpretation, limited accessibility of advanced testing, workflow integration challenges, and ethical and privacy concerns. Addressing these issues is essential to ensure equitable and clinically meaningful application of genomic technologies.

Future development of genomic cardiology will depend on expanding diverse genomic databases, improving clinical translation frameworks, and advancing emerging therapeutic approaches such as RNA‐based therapies and gene editing technologies. Although challenges remain, genomic cardiology holds strong potential to improve prevention, diagnosis, and treatment of cardiovascular diseases, with the greatest current impact observed in monogenic inherited cardiac conditions and guideline‐supported pharmacogenomic applications.

## Author Contributions

**Samira Kalayinia:** conceptualization, writing – review and editing. **Shiva Esmaei:** conceptualization, writing original draft preparation, writing – review and editing. **Neda Mohsen‐Pour:** conceptualization, writing – review and editing, writing original draft preparation. **Majid Maleki:** writing – review and editing. **Ali Sharafi:** writing, review, and editing. **Aydin Feyzi:** Figure preparation and illustration. All authors have read and agreed to the published version of the manuscript.

## Funding

The authors have nothing to report.

## Ethics Statement

The study complies with the Declaration of Helsinki and protocol was approved by the Ethics Committee of Rajaie Cardiovascular Institute, Tehran, Iran (IR.RHC.REC.1404.017).

## Consent

The authors have nothing to report.

## Conflicts of Interest

The authors declare no conflicts of interest.

## Data Availability

Data sharing is not applicable to this article as no new data were created or analyzed in this study.
